# Muscle mass and intramuscular fat of the quadriceps are related to muscle strength in non-ambulatory chronic stroke survivors: A cross-sectional study

**DOI:** 10.1371/journal.pone.0201789

**Published:** 2018-08-02

**Authors:** Naoki Akazawa, Kazuhiro Harada, Naomi Okawa, Kimiyuki Tamura, Hideki Moriyama

**Affiliations:** 1 Department of Physical Therapy, Faculty of Health and Welfare, Tokushima Bunri University, Tokushima, Tokushima, Japan; 2 Department of Physical Therapy, Faculty of Health, Medical care, and Welfare, Kibi International University, Takahashi, Okayama, Japan; 3 Department of Rehabilitation, Kasei Tamura Hospital, Wakayama, Wakayama, Japan; 4 Life and Medical Sciences Area, Health Sciences Discipline, Kobe University, Kobe, Hyogo, Japan; Ehime University Graduate School of Medicine, JAPAN

## Abstract

**Objective:**

Improving muscle mass and intramuscular fat in the mid-thigh increases the muscle strength of the paretic and non-paretic limbs in ambulatory chronic stroke survivors. There is a remarkable decrease in muscle mass and muscle strength and an increase in intramuscular fat in the quadriceps of both limbs of non-ambulatory compared with ambulatory survivors. Therefore, given that paretic lower extremity function does not recover sufficiently in the chronic phase, it may be helpful to improve muscle mass and intramuscular fat to increase muscle strength in the quadriceps of non-ambulatory chronic stroke survivors. However, these relationships remain unclear. The purpose of this study was to clarify the relationships between muscle strength, muscle mass, and intramuscular fat of the quadriceps in non-ambulatory chronic stroke survivors.

**Methods:**

**Study design:** A cross-sectional study.

**Participants:** Fifty non-ambulatory chronic stroke survivors.

**Main outcome measures:** Quadriceps muscle strength was measured using a handheld dynamometer. Transverse ultrasound images were acquired using B-mode ultrasound imaging. Muscle mass and intramuscular fat of the quadriceps were assessed based on muscle thickness and echo intensity, respectively.

**Data analysis:** Stepwise multiple regression analyses were used to identify the factors independently associated with the quadriceps muscle strength of the paretic and non-paretic limbs. To avoid multicollinearity, muscle thickness and echo intensity were entered into separate multiple regression models. Muscle thickness or echo intensity of the paretic or non-paretic limbs and other confounding factors were set as the independent variables.

**Results:**

Muscle thickness was positively related and echo intensity was negatively related to the quadriceps muscle strength of the paretic and non-paretic limbs.

**Conclusions:**

Muscle mass and intramuscular fat of the quadriceps are related to muscle strength in non-ambulatory chronic stroke survivors. Increasing muscle mass and decreasing intramuscular fat of the quadriceps of both limbs may improve muscle strength.

## Introduction

Enhancing the muscle strength of the paretic and non-paretic lower extremities in ambulatory chronic stroke survivors can improve gait ability [1–4]. The quadriceps muscle strength of chronic stroke survivors is also closely related to gait ability [4, 5]. Thus, increasing the muscle strength of the quadriceps is a main goal of physical therapy in these patients.

Recently, secondary changes in skeletal muscles, including decreases in muscle mass and increases in intramuscular fat, have been examined in ambulatory chronic stroke survivors [6–12], and less muscle mass and more intramuscular fat have been found in the paretic lower extremity than the non-paretic lower extremity [6, 7, 12]. In addition, improving muscle mass and intramuscular fat in the mid-thigh increases the leg press and leg extension strength of the paretic and non-paretic limbs in ambulatory chronic stroke survivors [13]. A more recent study [14] examined the muscle mass and intramuscular fat of the quadriceps of the paretic and non-paretic limbs in chronic stroke survivors by measuring muscle thickness and echo intensity in the ultrasound images, respectively. The results showed remarkably decreased muscle mass and increased intramuscular fat of the quadriceps of the paretic and non-paretic limbs in non-ambulatory chronic stroke survivors compared with those in ambulatory chronic stroke survivors [14]. Quadriceps muscle strength of the paretic and non-paretic limbs has also been shown to be lower in non-ambulatory than in ambulatory chronic stroke survivors [15]. Considering these findings, and given that the function of the paretic lower extremity does not recover sufficiently in the chronic phase [16], increasing muscle mass and decreasing intramuscular fat may be important for improving the muscle strength of the quadriceps in non-ambulatory chronic stroke survivors. However, the relationships among muscle mass, intramuscular fat, and muscle strength of the quadriceps of the paretic and non-paretic limbs in non-ambulatory chronic stroke survivors remain unclear. Understanding these relationships is important in selecting an approach for improving muscle mass, intramuscular fat, and muscle strength of the quadriceps in non-ambulatory chronic stroke survivors.

The aim of this study was to clarify the relationships between muscle strength, muscle mass, and intramuscular fat of the quadriceps on the paretic and non-paretic limbs in non-ambulatory chronic stroke survivors.

## Materials and methods

### Study design and participants

Participants were recruited to this cross-sectional study using advertisements. Fifty non-ambulatory chronic stroke survivors living in the community participated in the study. The inclusion criteria were >6 months since the stroke and inability to walk independently (defined as having a Functional Independence Measure [FIM] gait score [17] of 1–5). Prospective participants with a history of dementia or aphasia were excluded from the study. Table 1 shows the participants’ characteristics.

**Table 1:** Participant characteristics (n = 50).

Age, years, mean (SD)	78.7 (9.0)
Sex (male, female), n (%)	24 (48), 26 (52)
Weight, kg, mean (SD)	50.1 (11.2)
Height, cm, mean (SD)	154.5 (9.1)
Body mass index, kg/m^2^, median (IQR)	20.5 (18.6–22.4)
Type of stroke (cerebral hemorrhage, cerebral infarction), n (%)	18 (36), 32 (64)
Fugl–Meyer Assessment lower extremity score, median (IQR)	20.0 (16.8–28.0)
Functional Independence Measure gait score, median (IQR)	3.0 (1.0–4.0)
Time since stroke, month, median (IQR)	49.5 (13.8–122.8)
Quadriceps muscle strength, paretic limb, Nm, median (IQR)	15.6 (6.0–26.1)
Quadriceps muscle strength, non-paretic limb, Nm, median (IQR)	33.4 (21.3–43.6)
Quadriceps muscle thickness, paretic limb, mm, mean (SD)	15.5 (5.4)
Quadriceps muscle thickness, non-paretic limb, mm, mean (SD)	17.1 (6.2)
Quadriceps echo intensity, paretic limb (0–255)*, mean (SD)	79.6 (24.2)
Quadriceps echo intensity, non-paretic limb (0–255)*, mean (SD)	71.5 (25.2)

SD, Standard deviation. IQR, Interquartile range

*gray-scale.

### Ethical considerations

The study objectives and procedure were explained to the participant, and each participant who understood them and agreed to participate in this study provided written informed consent prior to participation. The study protocol and consent procedures were approved by the ethics committee of Kibi International University (No, 14–29).

### Outcome measurements

Primary outcomes were muscle strength, muscle mass, and intramuscular fat of the quadriceps of the paretic and non-paretic limbs. We also recorded the age, sex, weight, height, body mass index (BMI), Fugl–Meyer assessment (FMA) lower extremity score [18] (an index of paretic lower extremity function), FIM gait score, type of stroke, and time since stroke.

### Quadriceps muscle strength measurement

Isometric quadriceps muscle strength was measured using a handheld dynamometer (micro FET2; Hoggan Health Industries, Salt Lake City, UT, USA), which was placed just proximal to the ankle on the anterior surface of the leg while the participant was seated with knees and hip flexed to 90°, and the non-paretic hand was placed on a treatment table. We asked the participants to produce the maximum isometric quadriceps contraction for 3 to 4 s. The same physical therapist performed two measurements. Before testing, a physical therapist demonstrated the muscle strength measurement for the participants, who then performed two rehearsals to familiarize themselves with the procedure. The highest value was used to calculate the quadriceps torque by multiplying the strength (N) by the length of the lever arm (m). A previous study [19] reported high test-retest reliability (r = 0.98) of isometric quadriceps muscle strength measurement using a handheld dynamometer in patients with neurological disease. To confirm the reliability of the quadriceps muscle strength measurement of the paretic and non-paretic limbs in non-ambulatory chronic stroke survivors (4 males and 4 females, mean age 75.4 [standard deviation 11.0] years), we compared these measurements taken 1 week apart.

### Quadriceps muscle mass, intramuscular fat, and thigh subcutaneous fat mass measurements

Transverse ultrasound images were acquired using B-mode ultrasound imaging (Nanomaxx, SonoSite Japan, Tokyo, Japan) with a linear-array probe (L25n/13-6 MHz). The muscle mass and intramuscular fat content of the rectus femoris and vastus intermedius were assessed based on muscle thickness (MT) and echo intensity (EI), respectively [14, 20–30]. The validity of these muscle mass and intramuscular fat measurements derived from ultrasound images was proven in recent studies using magnetic resonance imaging [25, 26, 31]. Images of the rectus femoris and vastus intermedius were obtained at 30% of the distance from the anterosuperior iliac spine to the proximal end of the patella [14, 22, 24, 27]. The participants were positioned in a supine position with their lower extremities relaxed. Water-soluble transmission gel was then applied to the skin surface of the thigh, and the probe was pressed lightly against the skin to avoid deformation of the muscle. All images were obtained by the same investigator.

MT was determined as the distance between the superficial adipose tissue–muscle interface and the deep muscle–muscle interface for the rectus femoris [14]. MT of the vastus intermedius was measured as the distance between the superficial muscle–muscle interface and the bone–muscle interface [14]. EI was measured in regions of interest that were selected to include as much muscle as possible while avoiding bone and surrounding fascia [14, 20–30]. MT and EI values were then calculated using ImageJ 1.49 software (National Institutes of Health, Bethesda, MD, USA) [14, 20, 21, 23–29]. The EI was determined by computer-assisted 8-bit gray-scale analysis. The mean EI of the regions of interest was expressed as a value between 0 (black) and 255 (white) [14, 20, 21, 23–30]. Higher EI values indicate greater amounts of intramuscular fat [25, 26]. The sum of the MT of the rectus femoris and vastus intermedius was considered an indicator of quadriceps muscle mass. The EI of the quadriceps was calculated as the mean EI of the rectus femoris and vastus intermedius. Measurements of the MT and EI of the quadriceps of the paretic and non-paretic limbs in chronic stroke survivors have been shown to have high reliability (intraclass correlation coefficient [1.1] = 0.86 to 0.96) [14].

### FMA lower extremity score assessments

The FMA lower extremity score assesses movement, coordination, and reflexes. It has 17 items (maximum score 34) [18]. Each item is graded on a 3-point scale (0, cannot perform; 1, can partially perform; 2, can perform fully).

### FIM gait score assessments

The FIM gait score ranges from 1 (total assistance) to 7 (complete independence), and the level of physical assistance required for walking is represented by FIM gait scores 1–5 [17].

### Statistical analysis

All statistical analyses were conducted using SPSS version 24 software (IBM SPSS Japan, Tokyo, Japan). Variables were assessed for normality using the Shapiro–Wilk test. Parametric data are reported as the mean (standard deviation), whereas nonparametric data are expressed as the median (interquartile range). The relationships between quadriceps muscle strength of the paretic or non-paretic limbs and MT, EI, age, sex, BMI, time since stroke, and FMA lower extremity score were assessed using Kendall’s tau rank correlation coefficient. Stepwise multiple regression analyses were used to identify the factors that were independently associated with quadriceps muscle strength of the paretic and non-paretic limbs. We entered quadriceps MT and ET into another stepwise multiple regression model, respectively, because a pre-analysis revealed high correlation coefficients between the MT and ET of the quadriceps of both the paretic (r = −0.83) and the non-paretic (r = −0.81) limbs. For quadriceps muscle strength of the paretic limb, MT or EI, age, sex, BMI, time since stroke, and FMA lower extremity score were set as the independent variables. The same independent variables (except FMA lower extremity score) were included in the model with quadriceps muscle strength of the non-paretic limb as the dependent variable. In the correlation and stepwise multiple regression analyses, males and females were coded as 1 and 2, respectively. A value of *P* < 0.05 was considered to indicate statistical significance. In addition, we calculated the intraclass correlation coefficients (1.1) of the quadriceps muscle strength measurements.

### Sample size calculation

A previous study [30] reported an independent association (R^2^ = 0.29) between the MT and EI of the quadriceps and quadriceps muscle strength in middle-aged and elderly women. We therefore set the expected R^2^ of the multiple regression analysis in this study at 0.29. The effect size (f^2^) was calculated by the equation R^2^/ (1-R^2^) [32]. An a priori sample size calculation with f^2^ of 0.41, power of 0.90, alpha error of 0.05, and 5 to 6 predictor variables indicated that a sample size of at least 47 to 50 participants would be required. The sample size calculation was conducted using G* Power version 3.1.9.2 (Heinrich-Heine-Universität Düsseldorf, Germany).

## Results

**Fig 1:**
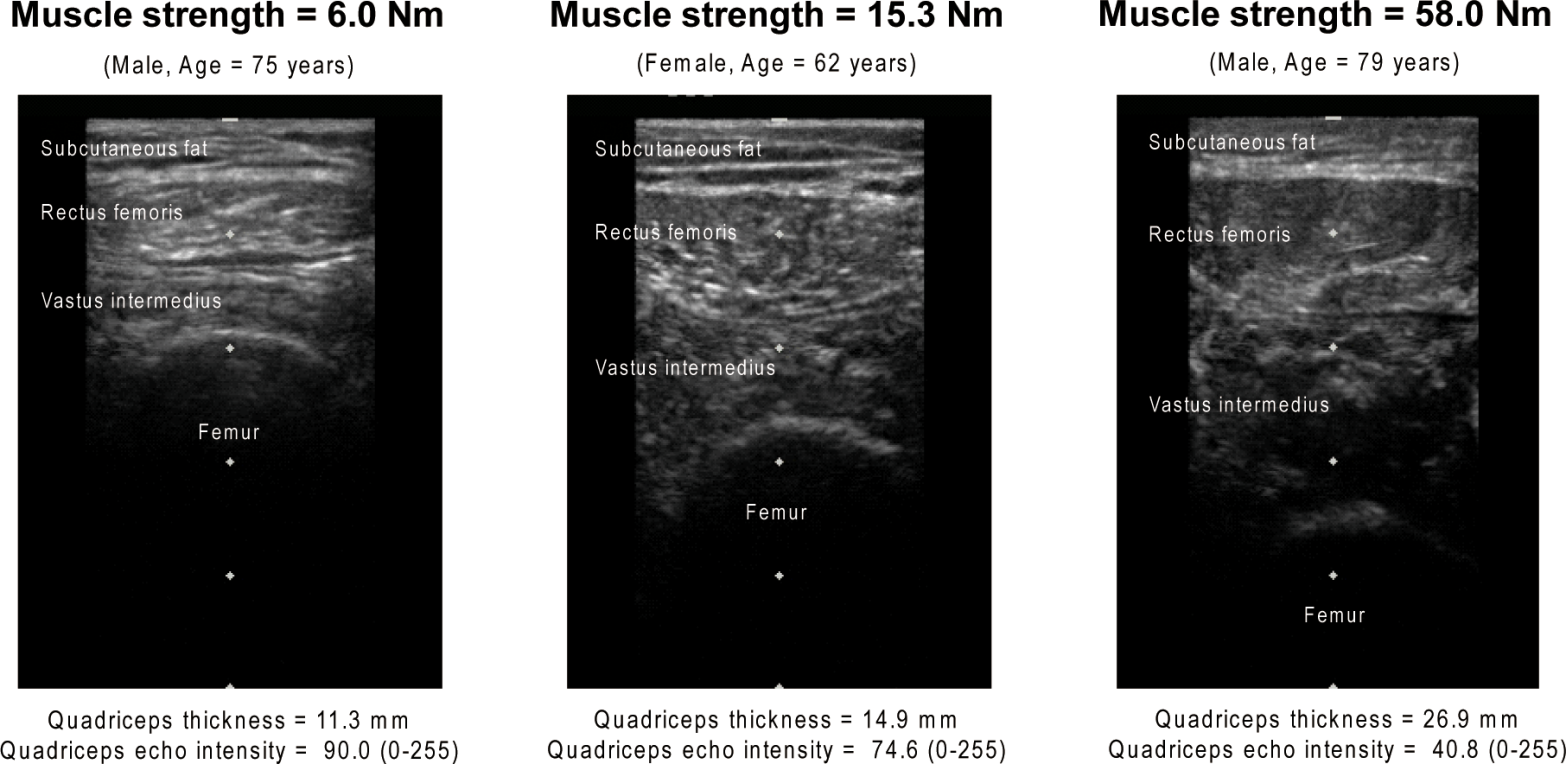
Typical ultrasound images of the paretic lower extremity.

Typical ultrasound images of the paretic lower extremity are shown in Fig 1. Tables 2 and 3 show the correlation coefficients between the quadriceps muscle strength of the paretic or non-paretic limbs and each variable. The quadriceps muscle strength of the paretic limb was significantly associated with FMA lower extremity score (Tau-b = 0.449), MT (Tau-b = 0.275), and EI (Tau-b = −0.247) (Table 2). In the non-paretic limb, quadriceps muscle strength was significantly related to age (Tau-b = −0.200), sex (Tau-b = −0.305), MT (Tau-b = 0.422), and EI (Tau-b = −0.372) (Table 3). The results of the stepwise multiple regression analyses are shown in Tables 4–7. There was no multicollinearity between the independent variables in the stepwise multiple regression analyses, and the variance inflation factors ranged from 1.000 to 1.869. MT (β = 0.43) and EI (β = −0.42), FMA lower extremity score (β = 0.58 and 0.50), and age (β = 0.21 and 0.29) were significantly independently associated with the quadriceps muscle strength of the paretic limb (Tables 4 and 5). MT (β = 0.75), BMI (β = −0.29) and EI (β = −0.50) were significantly independently associated with quadriceps muscle strength of the non-paretic limb (Tables 6 and 7). The intraclass correlation coefficients (1.1) of the quadriceps muscle strength measurements of the paretic and non-paretic limbs were 0.97 and 0.92, respectively.

**Table 2:** Correlation coefficients between quadriceps muscle strength of the paretic limb and each variable.

Variable	Correlation coefficient with quadriceps muscle strength of the paretic limb (Kendall’s Tau-b)	p value
Age	0.159	0.111
Sex	−0.211	0.077
Body mass index	−0.050	0.615
Fugl–Meyer Assessment lower extremity score	0.449	< 0.001
Time since stroke	−0.033	0.737
Quadriceps muscle thickness, paretic limb	0.275	0.005
Quadriceps echo intensity, paretic limb	−0.247	0.012

**Table 3:** Correlation coefficients between quadriceps muscle strength of the non-paretic limb and each variable.

Variable	Correlation coefficient with quadriceps muscle strength of the non-paretic limb (Kendall’s Tau-b)	p value
Age	−0.200	0.043
Sex	−0.305	0.010
Body mass index	0.125	0.203
Time since stroke	−0.097	0.323
Quadriceps muscle thickness, non-paretic limb	0.422	< 0.001
Quadriceps echo intensity, non-paretic limb	−0.372	< 0.001

**Table 4:** Stepwise multiple regression analysis (muscle thickness entry model) for muscle strength of the quadriceps on the paretic limb (R^2^ = 0.57).

	Partial regression coefficient	Standard error	95% Confidence interval of partial regression coefficient	Standardized partial regression coefficient	Variance inflation factor	p value
Fugl–Meyer Assessment lower extremity score	0.98	0.17	0.64, 1.32	0.58	1.03	< 0.001
Quadriceps muscle thickness, paretic limb	1.08	0.24	0.59, 1.57	0.43	1.03	< 0.001
Age	0.31	0.15	0.01, 0.61	0.21	1.06	0.044

**Table 5:** Stepwise multiple regression analysis (echo intensity entry model) for muscle strength of the quadriceps on the paretic limb (R^2^ = 0.54).

	Partial regression coefficient	Standard error	95% Confidence interval of partial regression coefficient	Standardized partial regression coefficient	Variance inflation factor	p value
Fugl–Meyer Assessment lower extremity score	0.85	0.18	0.50, 1.21	0.50	1.08	< 0.001
Quadriceps echo intensity, paretic limb	−0.24	0.06	−0.36, −0.11	−0.42	1.17	< 0.001
Age	0.44	0.16	0.10, 0.77	0.29	1.19	0.011

**Table 6:** Stepwise multiple regression analysis (muscle thickness entry model) for muscle strength of the quadriceps on the non-paretic limb (R^2^ = 0.40).

	Partial regression coefficient	Standard error	95% Confidence interval of partial regression coefficient	Standardized partial regression coefficient	Variance inflation factor	p value
Quadriceps muscle thickness, non-paretic limb	2.10	0.39	1.32, 2.88	0.75	1.49	< 0.001
Body mass index	−1.50	0.72	−2.96, −0.05	−0.29	1.49	0.043

**Table 7:** Stepwise multiple regression analysis (echo intensity entry model) for muscle strength of the quadriceps on the non-paretic limb (R^2^ = 0.24).

	Partial regression coefficient	Standard error	95% Confidence interval of partial regression coefficient	Standardized partial regression coefficient	Variance inflation factor	p value
Quadriceps echo intensity, non-paretic limb	−0.34	0.09	−0.51, −0.17	−0.50	1.00	< 0.001

## Discussion

Our results show that the muscle mass and intramuscular fat of the quadriceps are independently related to the muscle strength of the paretic and non-paretic limbs in non-ambulatory chronic stroke survivors. Improving the muscle mass and intramuscular fat of the mid-thigh has been shown to increase the leg press and leg extension strength of the paretic and non-paretic limbs in ambulatory chronic stroke survivors [13]. In addition, non-ambulatory chronic stroke survivors have decreased muscle mass and increased intramuscular fat of the quadriceps compared with ambulatory survivors [14]. The quadriceps muscle strength of the paretic and non-paretic limbs in non-ambulatory chronic stroke survivors is also lower than that of ambulatory survivors [15]. In other words, although the quadriceps of both limbs in non-ambulatory chronic stroke survivors show remarkably decreased muscle mass and muscle strength and increased intramuscular fat compared with ambulatory survivors, our findings suggest that the relationships among muscle mass, intramuscular fat, and muscle strength are similar among the two groups of patients.

Paretic lower extremity function does not recover sufficiently in the chronic phase [16]. We therefore speculate that an exercise-based intervention to increase muscle mass and reduce intramuscular fat might increase the muscle strength of the quadriceps of both the paretic and non-paretic limbs in non-ambulatory chronic stroke survivors. Resistance training for ambulatory chronic stroke survivors improved the muscle mass, intramuscular fat, and muscle strength of the paretic and non-paretic lower extremities [13]. However, although resistance training may be effective, it is sometimes difficult for non-ambulatory chronic stroke survivors. A previous study [33] reported that a physical activity intervention prevented both the age-associated increase in intramuscular fat of the mid-thigh and loss of muscle strength of the quadriceps in sedentary older adults. A more recent study [34] reported that physical activity improved the muscle mass, intramuscular fat, and muscle strength of the lower extremity in mobility-limited (Short Physical Performance Battery ≤ 9) older adults, and nutritional supplementation (whey protein and vitamin D) led to further reduction of intramuscular fat. Furthermore, neuromuscular electrical stimulation preserves quadriceps muscle mass in moderate or severe acute stroke patients [35]. Thus, the muscle mass, intramuscular fat, and muscle strength of the quadriceps in non-ambulatory chronic stroke survivors may be improved by a physical activity intervention combined with nutritional supplementation and neuromuscular electrical stimulation.

Decreased muscle mass and increased intramuscular fat in ambulatory chronic stroke survivors are associated not only with a decrease in muscle strength but also a decrease in aerobic exercise capacity and an increase in insulin resistance [6, 36]. These secondary changes in the skeletal muscle of ambulatory chronic stroke survivors lead to inactivity and consequently the pathogenesis and aggravation of diabetes [6, 9, 36]. We consider that intervention to increase muscle mass and decrease intramuscular fat in non-ambulatory chronic stroke survivors would not only improve their muscle strength but also lower the risk of harmful consequences resulting from secondary changes to the skeletal muscle.

This study has several limitations. First, although magnetic resonance imaging and computed tomography provide more accurate measurements of muscle mass and intramuscular fat, we used ultrasound to assess these parameters because it is easily accessible, quick to execute, and inexpensive [37, 38]. In addition, the validity of ultrasound for such measurements has been recently demonstrated in studies using muscle biopsy [39] and magnetic resonance imaging [25, 26, 31] as gold standards. Second, this study recruited chronic stroke survivors. Therefore, whether muscle mass and intramuscular fat in acute and non-ambulatory convalescent stroke survivors are related to the muscle strength of the quadriceps remains unclear. Third, this study examined patients’ quadriceps. Examining other muscles may produce different findings. Fourth, although spasticity could influence the relationship between muscle strength and muscle mass in chronic stroke survivors, we were unable to examine this influence in the current study. Finally, because of the cross-sectional study design, we were unable to clarify the causal relationship between quadriceps muscle strength, muscle mass, and intramuscular fat of the paretic and non-paretic limbs in non-ambulatory chronic stroke survivors. Thus, a further study to reveal the underlying nature of this relationship is warranted.

## Conclusions

Our findings suggest that muscle mass and intramuscular fat of the quadriceps are related to muscle strength in non-ambulatory chronic stroke survivors. An intervention combining physical activity with nutritional supplementation and neuromuscular electrical stimulation may be an effective approach for improving quadriceps muscle mass, intramuscular fat, and muscle strength in non-ambulatory chronic stroke survivors. Further studies are needed to reveal the causal relationship among them.
